# Comparative Analysis of Enamel Surface Roughness Between Herbal and Non-herbal Dentifrices: An In Vitro Study

**DOI:** 10.7759/cureus.80436

**Published:** 2025-03-11

**Authors:** Ramandeep Kaur, Arun Dodamani, Swapnali Patil, Sujata Chhabile, Girija Dodamani, Prashanth Vishwakarma, Seema Gupta

**Affiliations:** 1 Department of Public Health Dentistry, Sri Sukhmani Dental College and Hospital, Derabassi, IND; 2 Department of Public Health Dentistry, Jawahar Medical Foundation's Annasaheb Chudaman Patil Memorial Dental College, Dhule, IND; 3 Department of Prosthodontics, Jawahar Medical Foundation's Annasaheb Chudaman Patil Memorial Dental College, Dhule, IND; 4 Department of Orthodontics, Kothiwal Dental College and Research Centre, Moradabad, IND

**Keywords:** abrasive, enamel, herbal toothpaste, profilometer, surface roughness

## Abstract

Introduction: Toothbrushing is a primary contributor to the mechanical abrasion of enamel, and the degree of surface roughness is largely influenced by the abrasive components present in toothpaste and tooth powders. The abrasivity of dentifrices is determined by the type, composition, and particle size of the abrasive agents, which vary significantly between herbal and non-herbal formulations. The present study aimed to comparatively assess the surface roughness of enamel after brushing with herbal and non-herbal toothpastes, providing insights into their relative abrasivity and potential effects on long-term enamel wear.

Materials and methods: A total of 48 extracted premolar teeth were collected and randomly assigned into six groups. The groups included the following: Group 1 (n=8), a non-herbal toothpaste (Colgate Max Fresh, Colgate-Palmolive, New York, New York, United States); Group 2 (n=8), a non-herbal toothpaste (Pepsodent, Unilever, London, United Kingdom); Group 3 (n=8), a herbal toothpaste (Himalaya Complete Care, Himalaya Wellness Company, Karnataka, India); Group 4 (n=8), a herbal toothpaste (Dabur Meswak, Dabur India Ltd., New Delhi, India); Group 5 (n=8), a herbal tooth powder (Vicco, Vicco Labs, Maharashtra, India); and Group 6 (n=8), the control group using only distilled water. The surface roughness of the enamel in all groups was evaluated both at baseline (pre-brushing) and after a 28-day brushing regimen. Data analysis was performed using a paired t-test for intragroup comparisons and an analysis of variance (ANOVA) for intergroup comparisons to assess statistical significance.

Results: Statistical analysis demonstrated no significant differences in the surface roughness between the pre- and post-brushing measurements in any of the experimental groups. The baseline (pre-brushing) surface roughness values did not show any statistically significant variation among the groups (p=0.145), indicating initial homogeneity. However, following the 28-day brushing period, a statistically significant difference in the mean increase in surface roughness after post-brushing was observed among the groups (p=0.032), suggesting that the abrasive effects of the different dentifrices varied, leading to measurable differences in enamel roughness. Vicco tooth powder caused the highest increase in surface roughness, while Colgate Max Fresh led to the lowest increase.

Conclusion: Our study highlights that while non-herbal pastes and herbal powders exhibit differential effects on enamel roughness, all tested formulations remained within safe limits for regular use. However, the significant increase in roughness with herbal tooth powder emphasizes the need for further formulation optimization to balance efficacy and enamel safety.

## Introduction

Effective plaque control is fundamental for maintaining oral health, as dental plaque is the primary etiological factor in the development of dental caries and periodontal diseases [1]. A toothbrush is the principal instrument for plaque removal and plays a crucial role in the prevention and maintenance of oral hygiene [2]. Toothpaste functions as an abrasive agent that facilitates the removal of dental plaque and food debris, aids in halitosis suppression, and delivers active ingredients that help prevent tooth decay and periodontal diseases, thereby contributing to overall oral health [3].

Although the combined use of a toothbrush and toothpaste enhances the mechanical removal of plaque, improper brushing techniques have been associated with detrimental effects on dental structures beyond plaque control [4]. The term abrasion refers to the wear induced by toothpastes, toothbrushes, and polishing pastes. The abrasiveness of toothpaste is influenced by several factors, including the concentration of abrasive materials, particle size, the surface characteristics of abrasive particles, and the chemical composition of the dentifrice [5]. Moreover, chronic toothbrushing with abrasive dentifrices has been implicated as a contributory factor in gingival recession and tooth wear, although the precise mechanism by which abrasion varies with different toothbrush types and dentifrice formulations remains unclear [6]. Several in vitro studies have employed profilometric analysis to assess surface abrasivity, where a profilometer quantitatively evaluates changes in surface roughness [7]. This device functions by generating surface tracing using digital and analog systems and subsequently calculating the mean surface roughness.

Additionally, chemicals such as triclosan and chlorhexidine have been incorporated into mouthrinses and dentifrices to prevent plaque formation and gingivitis [3]. However, these compounds have been associated with adverse effects, including tooth discoloration and altered taste perception [8]. These concerns have led to increased emphasis on incorporating natural ingredients into herbal toothpaste formulations [9]. The current shift in consumer preference for alternative medicinal practices, particularly herbal-based oral care products, has resulted in a growing interest in herbal toothpaste because of its purported benefits for dental health maintenance. Despite its increasing popularity, limited research exists regarding the potential enamel-damaging effects of herbal toothpastes owing to their abrasive nature. Therefore, the primary aim of the present study was to compare and evaluate the abrasive effects of herbal and non-herbal toothpastes on extracted human permanent teeth. The secondary aim was to evaluate the hardness of the enamel surface and correlate it with surface roughness.

## Materials and methods

Study design

This in vitro study was conducted at Jawahar Medical Foundation's Annasaheb Chudaman Patil Memorial Dental College, Dhule, from October 2023 to March 2024. It was approved by the institute's Institutional Ethics Committee (approval number: EC/NEW/INST/2022/2959/013) and followed the principles of the Declaration of Helsinki. Written informed consent was obtained from all patients for the use of extracted teeth. 

Inclusion and exclusion criteria

The extracted human premolars obtained for orthodontic or therapeutic reasons were used in this study. Teeth with intact enamel surfaces free from caries, restorations, fractures, or structural defects were included after initial screening using stereomicroscopy at 40× magnification. Specimens exhibiting pre-existing surface irregularities greater than 10 µm were excluded to maintain baseline uniformity.

Sample size estimation

Sample size estimation was conducted using G*Power Version 3.2.9 (Heinrich-Heine-Universität Düsseldorf, Düsseldorf, Germany) to achieve a statistical power of 95% with a significance level (alpha error) of 5%. Based on a minimum effect size of 1.75, derived from a prior study by Korsuwannawong et al. [10], a total sample size of 48 teeth (eight per group) was determined to be adequate. The referenced study evaluated the enamel surface roughness of herbal and non-herbal toothpaste and reported a mean difference of 0.02 (0.049-0.071), with a pooled standard deviation of 0.035.

Methodology

A total of 48 extracted teeth were randomly assigned to six groups: Group 1 (n=8), a non-herbal toothpaste (Colgate Max Fresh, Colgate-Palmolive, New York, New York, United States); Group 2 (n=8), a non-herbal toothpaste (Pepsodent, Unilever, London, United Kingdom); Group 3 (n=8), a herbal toothpaste (Himalaya Complete Care, Himalaya Wellness Company, Karnataka, India); Group 4 (n=8), a herbal toothpaste (Dabur Meswak, Dabur India Ltd., New Delhi, India); Group 5 (n=8), a herbal tooth powder (Vicco, Vicco Labs, Maharashtra, India); and Group 6 (n=8), the control group using only distilled water. The composition of the toothpastes used in this study is listed in Table 1.

**Table 1:** Composition of the different toothpastes used in the study. Group 6 is using distilled water without dentifrice. RDA: relative dentin abrasivity

Group	Brand name	Nature	RDA value	Composition
Group 1	Colgate Max Fresh (Colgate-Palmolive, New York, New York, United States)	Non-herbal paste	83	Calcium carbonate, sorbitol, sodium lauryl sulfate, silica, titanium dioxide, sodium silicate, flavor, sodium monofluorophosphate, sodium bicarbonate, potassium nitrate, benzyl alcohol, sodium saccharin, limonene
Group 2	Pepsodent (Unilever, London, United Kingdom)	Non-herbal paste	150	Sorbitol, hydrated silica, sodium lauryl sulfate, PEG-32, zinc citrate, calcium carbonate, hydrated silica
Group 3	Himalaya Complete Care (Himalaya Wellness Company, Karnataka, India)	Herbal paste	70	Water, calcium carbonate, xylitol, silica, lauryl glucoside, *Stevia rebaudiana*, sodium bicarbonate, menthol, sodium chloride, thymol, *Melia azadirachta* leaf extract, *Punica granatum* fruit extract, *Embelia ribes* fruit extract, *Phyllanthus emblica* fruit extract, *Terminalia chebula* fruit extract, *Acacia arabica* bark extract
Group 4	Dabur Meswak (Dabur India Ltd., New Delhi, India)	Herbal paste	140	Calcium carbonate, water, sorbitol, sodium lauryl sulfate, silica, flavor, carrageenan, sodium silicate, sodium saccharin, cellulose gum and clove oil, lavang oil, pudina satva, tomar beej, sunthi, karpura, pippali, and garlic
Group 5	Vicco (Vicco Labs, Maharashtra, India)	Herbal powder	16.52	Bakul, Manjishtha, Patang, Kabab-Chini, Babhul, Khair, Jeshthamadh, Jambhul, Bor, Amla, Harda, Behada, Vajradanti, Anantmul, Akkalkadha, Dalchini, Lavang, Acrod, Maifal, Ajwain

The teeth were positioned within a mold measuring 20×15×10 mm, ensuring that the buccal surfaces of the teeth were oriented upward. Vinyl polysiloxane (Aquasil Ultra, Dentsply Sirona, New York, United States) was subsequently poured into the mold until a diameter of 5 mm of the buccal surface protruded above the contour height of the teeth. A powered, rechargeable electric toothbrush (Oral-B, Braun GmbH, Kronberg, Germany) was used in this study. The toothbrush functioned at a frequency of 8,800 oscillations per minute and 20,000 pulsations per minute. To ensure methodological consistency, the toothbrush head was securely stabilized and positioned directly over the specimens, thereby maintaining a continuous and uniform contact throughout the brushing procedure. A separate toothbrush was placed in each subgroup to mitigate the risk of cross-contamination. Brushing was conducted twice daily for 60 seconds per specimen, with a 12-hour interval separating each brushing session twice daily. A 1:3 slurry of toothpaste or tooth powder was mixed with artificial saliva at 37°C and used for brushing. The brushing regimen was conducted over a 28-day period to simulate real-world conditions. Between the brushing sessions, the specimens were stored in artificial saliva with a controlled pH of 6.8-7.2 to maintain enamel hydration and prevent dehydration-related artifacts [11].

The baseline surface roughness (Ra values in micrometers as Ra in µm) was assessed using contact-type surface profilometry (Talysurf Series 2, Taylor Hobson Ltd., Leicester, England). Following the intervention, final surface roughness measurements were obtained at six different locations per sample, and the mean value was used for statistical analysis.

Statistical analysis

Statistical analysis was conducted using IBM SPSS Statistics for Windows, Version 23.0 (Released 2015; IBM Corp., Armonk, New York, United States). The normality of the data was assessed using the Shapiro-Wilk test and further confirmed through a Q-Q plot, which indicated a normal distribution. Intragroup comparisons for pre- and post-brushing were analyzed with a paired t-test, and intergroup comparisons of surface roughness were performed with one-way analysis of variance (ANOVA) followed by post hoc analysis using the Tukey test. Intraclass correlation coefficient (ICC) analysis was conducted to assess consistency across the profilometric methods. Statistical significance was set at p<0.05, with Bonferroni correction applied for multiple comparisons to minimize the risk of type 1 errors.

## Results

The comparison of surface roughness before and after brushing with different toothpastes using the paired t-test revealed a statistically significant increase in roughness across all tested groups (p=0.001). Among all the groups, Group 4 (1.13±0.10 µm) showed the highest post-brushing surface roughness, followed by Group 5 (1.12±0.10 µm), Group 2 (1.11±0.11 µm), and Group 6 (1.10±0.10 µm). The least post-brushing surface roughness were noticed in Groups 1 (1.07±0.08 µm) and 3 (1.08±0.12 µm). The increased surface roughness in the control group showed that mechanical brushing also affected enamel surface texture. The findings also indicated that most of the tested toothpaste formulations contributed to increased surface roughness after brushing, with the Dabur Meswak paste creating the highest surface roughness. This finding suggests that toothpaste abrasivity varies across brands and potentially influences enamel wear and long-term dental health (Table 2).

**Table 2:** Comparison of surface roughness (Ra in µm) by the groups using paired t-test. Data are presented in mean and SD. SD: standard deviation; CI: confidence interval; Ra: roughness average *p<0.05: significant

Groups	N	Pre-brushing		Post-brushing		t stats	P-value
		95% CI	Mean±SD	95% CI	Mean±SD		
Group 1	8	0.85-0.95	0.9±0.06	1-1.14	1.07±0.08	5.51	0.001*
Group 2	8	0.77-0.93	0.85±0.09	1.02-1.2	1.11±0.11	6.51	0.001*
Group 3	8	0.82-0.92	0.87±0.06	0.99-1.18	1.08±0.12	5.32	0.001*
Group 4	8	0.83-0.93	0.88±0.06	1.04-1.21	1.13±0.10	5.74	0.001*
Group 5	8	0.74-0.89	0.81±0.09	1.04-1.2	1.12±0.10	9.53	0.001*
Group 6	8	0.88-0.97	0.92±0.05	1.01-1.19	1.10±0.10	6.53	0.001*

The intergroup comparison of surface roughness using ANOVA revealed no statistically significant difference in the pre-brushing (p=0.571) surface roughness values among the toothpaste groups, indicating that the initial surface roughness was comparable across all groups. However, the percentage increase in surface roughness showed a significant difference between the groups (p=0.032), suggesting that the different toothpaste formulations influenced enamel roughness to varying extents. Among the tested toothpastes, Group 5 exhibited the highest percentage increase in surface roughness, followed by Groups 2, 4, and 3. Groups 1 and 6 showed the least increase in surface roughness, indicating that Group 1 was comparable to the control group. These findings suggest that while pre- and post-brushing roughness levels were similar across the groups, the abrasivity of the toothpaste significantly influenced the degree of increase in enamel roughness. Toothpastes with higher abrasivity, such as Vicco and Pepsodent, may contribute to greater surface roughness, potentially affecting the enamel integrity over time (Table 3).

**Table 3:** Intergroup comparison of surface roughness (Ra in µm) by ANOVA test. Data are presented in mean and SD. SD: standard deviation; ANOVA: analysis of variance; Ra: roughness average *p<0.05: significant

Groups	Group 1	Group 2	Group 3	Group 4	Group 5	Group 6	F value	P-value
Pre-brushing	0.9±0.06	0.85±0.09	0.87±0.06	0.88±0.06	0.81±0.09	0.92±0.05	2.35	0.571
Post-brushing	1.07±0.08	1.11±0.11	1.08±0.12	1.13±0.10	1.12±0.10	1.1±0.10	0.39	0.857
% increase	19.06±10.19	31.83±16.09	24.66±13.39	28.3±14.81	38.51±13.83	19.02±8.03	2.73	0.032*

Post hoc analysis was conducted to assess pairwise comparisons of surface roughness increases among the different toothpaste groups. The results indicated that there were no statistically significant differences in the surface roughness between the toothpaste groups. However, a significant difference (p=0.049) was observed between Groups 5 and 6, suggesting that Vicco caused a significantly higher increase in surface roughness than the control group. Other toothpaste brands, including Colgate Max Fresh, Pepsodent, Himalaya Complete Care, and Dabur Meswak, did not show notable differences in their effects on enamel surface roughness when compared to one another (Table 4).

**Table 4:** Pairwise comparison of groups (p-values) by post hoc Tukey test. The numerical values in the table represent p-values. *p<0.05: significant

Pairwise groups	Group 1	Group 2	Group 3	Group 4	Group 5
Group 1	1	0.381	0.954	0.716	0.05
Group 2	0.381	1	0.878	0.994	0.907
Group 3	0.954	0.878	1	0.993	0.293
Group 4	0.716	0.994	0.993	1	0.623
Group 5	0.05	0.907	0.293	0.623	1
Group 6	1	0.377	0.952	0.712	0.049*

## Discussion

The surface roughness of enamel plays a crucial role in oral health and restorative dentistry by influencing plaque accumulation, bacterial adhesion, and the structural integrity of the enamel. Research suggests that enamel surfaces with a roughness average (Ra) greater than 0.2 µm significantly enhance bacterial colonization, which can contribute to dental plaque formation and periodontal diseases [12]. In our study, all tested groups showed values of less than 0.2 µm. Additionally, excessive enamel roughness can compromise the mechanical properties, potentially leading to microcrack propagation, reduced wear resistance, and increased susceptibility to erosion and staining [13]. Given these implications, understanding the impact of different dentifrices on enamel surface roughness is essential for evaluating their long-term effects on oral health.

The study further revealed that non-herbal toothpaste formulations, particularly Colgate Max Fresh, showed the least increase in enamel roughness which is also supported by a medium relative dentin abrasivity (RDA) value of 83, whereas Vicco tooth powder and Dabur Meswak exhibited the highest abrasivity, despite low RDA values for Vicco tooth powder. This could have been due to the fact that the RDA is contingent upon standardized brushing conditions utilizing radioactive dentin, which exhibits a softness superior to that of enamel. The formulations of Vicco tooth powder and Dabur Meswak may incorporate larger, irregular, or more abrasive particulates, potentially exacerbating enamel roughness despite possessing a comparatively lower RDA value. In contrast, Colgate Max Fresh, which possesses an RDA of 83, is likely to contain smaller, rounded silica abrasives that serve to polish enamel rather than induce roughening. These findings highlight the potential impact of toothpaste abrasivity on enamel integrity and emphasize the importance of formulation selection for optimal oral health maintenance. Colgate Max Fresh contains hydrated silica or calcium pyrophosphate, which are relatively softer and more controlled abrasives. Vicco and Dabur Meswak contain natural herbal or mineral-based abrasives such as calcium carbonate or clays, which have larger and harder particles that increase their abrasivity [14]. Colgate Max Fresh contains sodium lauryl sulfate, which helps with foaming but does not significantly contribute to abrasivity. Herbal tooth powders often rely on mechanical cleaning rather than detergents, leading to greater abrasion [15]. This finding was supported by previous studies [16,17], where non-herbal toothpastes showed less abrasiveness than herbal toothpastes.

Toothpaste serves as a mechanical aid for plaque removal and facilitates effective oral hygiene. However, the abrasive properties of dentifrices can lead to enamel surface wear, which has been widely discussed in the literature. The abrasiveness of a toothpaste formulation depends on several factors, including the type and concentration of abrasive agents, particle size, and chemical composition [5]. Although abrasives help remove extrinsic stains and plaque, excessive abrasivity can result in increased surface roughness, which in turn may lead to enamel erosion and dentin hypersensitivity [6].

The results of this study are consistent with prior research indicating that the selection of toothpaste significantly influences enamel surface texture [18]. The statistically significant increase in mean surface roughness values across all groups post-brushing underscores the impact of both mechanical brushing and the abrasivity of toothpaste. Herbal toothpastes have gained popularity because of their perceived natural benefits, including antimicrobial properties and reduced chemical additives. However, the abrasiveness of herbal toothpaste remains a topic of concern. The results of this study indicated that herbal toothpaste formulations demonstrate varied abrasivity, with some exhibiting significant enamel wear. Specifically, Vicco tooth powder and Dabur Meswak showed the highest percentage increase in surface roughness, whereas Himalaya Complete Care demonstrated relatively lower abrasivity. These findings align with studies suggesting that certain herbal toothpaste formulations may contain abrasive agents such as neem, salt, and clove, which can contribute to enamel wear [9].

Our results contradict those of a study by Aggarwal et al. [19], who reported lower surface roughness with herbal toothpastes than with non-herbal toothpastes. This could have been due to differences in methodology, as they did not mention the brushing method and standardization processes. Non-herbal toothpaste formulations, such as Colgate Max Fresh and Pepsodent, also exhibited abrasivity; however, Colgate Max Fresh showed the least increase in enamel roughness. This could be attributed to the controlled particle size and optimized abrasive composition of modern non-herbal dentifrices, which are formulated to balance plaque removal efficacy with enamel preservation [10]. The comparable surface roughness between the Colgate Max Fresh and control groups suggests that certain non-herbal toothpaste formulations may minimize enamel wear while maintaining effective cleaning properties.

The study findings demonstrated that even the control group, which used only distilled water, exhibited an increased enamel roughness after brushing. This suggests that the mechanical action of brushing itself contributes to enamel surface alterations, independent of toothpaste abrasivity. These results support previous studies indicating that brushing forces, frequency, and technique play crucial roles in enamel wear [6,20]. The use of an electric toothbrush in this study ensured standardized brushing conditions, thereby eliminating the variability associated with manual brushing. The high oscillation and pulsation rates of the electric toothbrush may have amplified the effect of abrasive agents in toothpaste formulations, further influencing the enamel roughness. Although electric toothbrushes are known to improve plaque removal efficiency, their prolonged use with highly abrasive toothpaste may accelerate enamel wear, warranting careful selection of toothpaste formulations [21].

The highest roughness observed in the Vicco and Dabur Meswak groups suggests that prolonged use of these formulations may lead to progressive enamel softening, thereby increasing the susceptibility to erosion and hypersensitivity. Previous studies have reported that high-abrasivity toothpaste can lead to microstructural alterations in the enamel, affecting its hardness and increasing the risk of mechanical wear over time [13]. Additionally, the absence of humectants and thickening agents in herbal powders may contribute to their higher abrasivity [10]. Non-herbal pastes contain glycerin or sorbitol, which help suspend abrasive particles uniformly, thereby minimizing direct frictional contact with the enamel. In contrast, herbal powders are applied in a dry or minimally hydrated state, leading to higher frictional forces against enamel surfaces [22].

Wiegand et al. [23] compared the brushing force between manual and electric toothbrushes and found that the average brushing force of the manual toothbrush (1.6±0.3 N) was significantly higher than that of the electric toothbrushes (0.9±0.2 N). The manual toothbrush caused the highest abrasion of sound and eroded dentin; therefore, patients with severe tooth wear and exposed and/or eroded dentin surfaces should use electric toothbrushes to reduce abrasion.

Clinical implications and recommendations

These findings have several clinical implications. First, consumers should be aware that toothpaste formulations vary in their abrasivity levels and that toothpaste selection should be based on individual oral health needs. For patients with enamel erosion, hypersensitivity, or gingival recession, low-abrasivity toothpaste may be preferable to minimize further enamel wear. Dental professionals should recommend toothpastes with controlled abrasivity to balance plaque removal and enamel preservation. Second, this study underscores the importance of brushing techniques in mitigating enamel wear. Patients should be educated on gentle brushing methods to avoid excessive pressure and prolonged brushing duration, particularly when using abrasive toothpaste formulations. Further research on herbal toothpaste formulations is warranted to explore alternative natural abrasives that provide effective cleaning and reduce enamel wear. Standardized testing protocols should be established to assess toothpaste abrasivity and ensure consumer safety and product efficacy.

Limitations

Although this study provides valuable insights into the abrasivity of different toothpaste formulations, several limitations of this study should be acknowledged. First, the in vitro design did not fully replicate the complex oral environment in which factors such as salivary composition, pH fluctuations, and dietary habits influence enamel wear. Future studies that incorporate in situ or clinical trials will provide more comprehensive findings. Second, the study focused on the short-term effects over a 28-day period. Long-term studies evaluating cumulative enamel wear for months or years would provide a clearer understanding of the potential risks associated with prolonged toothpaste use. Additionally, scanning electron microscopy (SEM) offers detailed visualization of enamel surface alterations, complementing profilometric measurements. Future research should explore the interplay between abrasivity and remineralization, particularly in fluoride-based and herbal toothpaste formulations, to determine their overall impact on enamel health.

## Conclusions

Our study highlights that, while non-herbal pastes and herbal powders exhibited differential effects on enamel roughness, all tested formulations remained within the safe limits for regular use. While all the tested toothpaste formulations contributed to increased enamel roughness post-brushing, certain herbal formulations exhibited higher abrasivity, potentially affecting enamel integrity over time. Non-herbal toothpastes, particularly Colgate Max Fresh, demonstrated lower abrasivity, making it a preferable option for enamel preservation. These findings emphasized the importance of toothpaste selection, brushing techniques, and continued research to optimize oral care products for long-term dental health.
